# Carbon Nanotube-Graphene Hybrid Electrodes with Enhanced Thermo-Electrochemical Cell Properties

**DOI:** 10.3390/nano9101450

**Published:** 2019-10-12

**Authors:** Yuqing Zhou, Weijin Qian, Weijun Huang, Boyang Liu, Hao Lin, Changkun Dong

**Affiliations:** Institute of Micro-Nano Structures & Optoelectronics, Wenzhou University, Wenzhou 325035, China; z857744841@163.com (Y.Z.); 18857757816@163.com (W.H.); wonderain@outlook.com (B.L.); linhao.good@icloud.com (H.L.)

**Keywords:** carbon nanotube, graphene, hybrid electrode, electrophoretic deposition, adhesion, thermo-electrochemical cell

## Abstract

Carbon nanotube-Graphene (CNT-Gr) hybrids were prepared on stainless steel substrates by the electrophoretic deposition (EPD) to make the thermo-electrochemical cell (TEC) electrodes. The as-obtained TEC electrodes were investigated by the SEM, XRD, Raman spectroscopy, tensile, and surface resistance tests. These hybrid electrodes exhibited significant improved TEC performances compared to the pristine CNT electrode. In addition, these hybrid electrodes could be optimized by tuning the contents of the graphene in the hybrids, and the CNT-Gr-0.1 hybrid electrode showed the best TEC performance with the current density of 62.8 A·m^−2^ and the power density of 1.15 W·m^−2^, 30.4% higher than the CNT electrode. The enhanced TEC performance is attributed to improvements in the electrical and thermal conductivities, as well as the adhesion between the CNT-Gr hybrid and the substrate. Meanwhile, the relative conversion efficiency of the TECs can reach 1.35%. The investigation suggests that the growth of CNT-Gr hybrid electrodes by the EPD technique may offer a promising approach for practical applications of the carbon nanomaterial-based TEC electrodes.

## 1. Introduction

The thermo-electrochemical cell (TECs) also called as thermo cell or thermogalvanic cell [1,2,3], converting directly the thermal energy to electrical energy, is an attractive renewable energy device for harvesting the low-grade heat (e.g., waste heat from industry and vehicles, the heat from storage or computer system) due to advantages of low cost, simple configuration, direct energy conversion, and stable operation [4,5].

The current (I) and the voltage (V_oc_) between the two working electrodes are two important parameters for the TECs. The relation between I and V_oc_ can be obtained by the equation: I = V_oc_/R, where V_oc_ represents the open-circuit potential, which is mainly determined by the temperature difference [6]. R is the internal resistance of the TEC, including four aspects, i.e., charge transfer, ohmic, solution diffusion, and thermal diffusion resistances [5,7]. The material with high electrical conductivity means lower charge transfer resistance. Similarly, the material with high thermal conductivity means lower thermal resistances, which can benefit for decreasing the temperature loss at the bodies of two electrodes, leading to higher energy conversation efficiency of the TEC [4,5,7]. Due to good electric and thermal conductivities, excellent chemical stability, and unique structural properties, carbon nanotubes (CNTs) showed promising properties in the TEC application [4,5,7,8,9,10,11,12,13,14], and the electrophoretic deposition (EPD) method was applied to fabricate CNT TECs for merits of process simplicity and large-scale capability [5,10,15]. The graphene, with priorities of large surface area, high electric/thermal conductivities, and high chemical/electrochemical stabilities, has been widely investigated in the energy storage and conversation applications [16,17,18,19,20]. Recently, CNT-based hybrid electrodes, i.e., the electrodes with at least two composition materials, were applied to fabricate TEC electrodes with improved energy conversation efficiency because of the synergetic effect between different materials [8,11,12,13]. Im et al. reported that the CNT-Pt hybrid nanostructure could achieve much higher conversation efficiency in comparison with the pristine CNTs for the faster kinetics and larger electroactive sites [12]. Our investigation showed that CNT-Ag hybrids exhibited better TEC performances due to higher electric and thermal conductivities, as well as more activation sites [11]. Romano et al. reported that the TEC performances of single-walled CNT-reduced graphene oxide hybrid electrodes were improved significantly compared to the pristine CNT sample, due to the enhancement of the mass transport property [13]. Despite this progress, the controllable synthesis of CNT-Graphene hybrids by the EPD technique to make the TEC electrode has not yet been reported, which is very significant for promoting practical applications of the CNT-based TECs.

In this study, the CNT-graphene hybrids were facilely fabricated by the EPD technique to make the TEC electrodes. These hybrid electrodes exhibit much improved TEC performances compared to the pristine CNT electrode. The TEC performances of CNT-Graphene hybrid electrodes were optimized by tuning the graphene content in the CNT-Graphene hybrids and the optimized TEC performance was obtained with the maximum current density of 62.8 A·m^−2^ and the power density of 1.15 W·m^−2^, 30.4% higher than the pristine CNT electrode. The enhanced TEC performance is attributed to the better electrical and thermal conductivities, as well as stronger adhesion between the CNT-Graphene hybrid electrode and the substrate. Meanwhile, the relative conversion efficiency of the TECs could reach 1.35%. This investigation suggests that facilely synthesis of CNT-Graphene hybrid electrodes by EPD technique provides a promising approach to develop practical CNT-based TEC devices.

## 2. Materials and Methods

### 2.1. Synthesis of the CNT-Graphene Hybrid Electrodes

**Figure 1:**
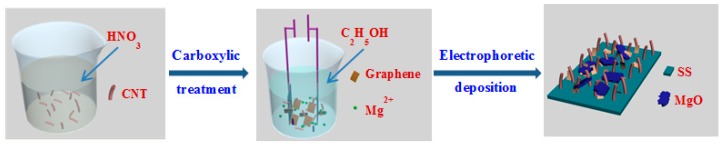
Production process of carbon nanotube CNT-Graphene hybrid.

The CNT-Graphene hybrids were synthesized on the stainless steel (SS) substrate by the EPD method. Firstly, the stainless-steel substrates and the multiwalled carbon nanotubes (MWNTs, Shenzhen Nanotech. Port Co. Ltd., Shenzhen, China) were ultrasonically cleaned and carboxylic pretreated, respectively, as described in our previous reports [5,10]. Secondly, the carboxylic CNTs, Graphene powder, and MgCl_2_ (99.5%, Aladdin) were added into ethanol solution and sonicated for about 2 h to form the stable suspension. The stainless-steel substrate and the counter electrode (the SS foil) were immersed into the suspension with a distance of 4 cm and the constant voltage of 120 V. Finally, the CNT-Graphene hybrid was annealed in the furnace at 700 °C with the duration of 1 h. The TEC performances of the CNT-Graphene hybrids were optimized by tuning the concentration of Graphene. The as-prepared hybrids are denominated as CNT-Graphene-x, where x is the concentration of the Graphene from 0 to 0.4 g L^−1^. The main synthesis processes of the CNT-Graphene hybrid are illustrated in Figure 1.

### 2.2. Characterization

The morphologies of the pristine CNTs and the CNT-based hybrids were observed using scanning electron microscopy (SEM, JEOL JSM-7100F). The species of all samples were identified by X-ray diffraction (XRD, GmbH SMART APEX with the X-ray source wavelength of 0.154056 nm) and Raman spectroscopy (Renishaw Invia Raman Microscope with the laser wavelength of 633 nm). To investigate the adhesion between the pristine CNT or the CNT-based hybrid and the SS substrates, tensile tests were performed by an Instron 3343 equipment [10]. Briefly, the sample was fixed with a clamp and then pasted with the double-sided tape. During the test, the CNT-based films were dragged by the tape until these films detached from the substrate. The surface resistances of all the electrodes were measured by four probe testers [10].

### 2.3. TEC Measurements

The CNT and the CNT-based hybrid electrodes were tested by the Cup-Shaped TEC equipment and the Cup-Shaped device of TEC and characterization of TEC performance were described in other reports and our previous investigations [4,5,11]. Briefly, two test electrodes were immersed in the potassium ferri/ferrocyanide (0.4 mol·L^−1^, Aladdin) aqueous solution, the temperatures of two test sides were controlled by the heating tape and ice water, respectively, and measured by OMEGA thermocouple probes. The TEC parameters, such as short-circuit current and the open-circuit potential, were acquired by the KEITHLEY 2440 multimeter (Tektronix Technology Co. Ltd., Beaverton, OR, USA).

## 3. Results and Discussion

### 3.1. Structure Characterization

**Figure 2:**
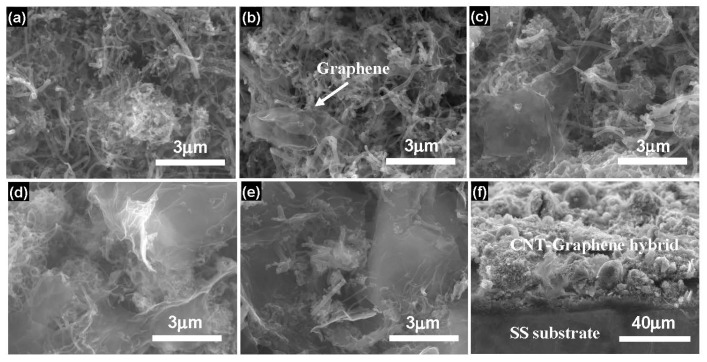
SEM images of the pristine CNTs and CNT-Graphene hybrids. (**a**) the pristine CNTs, (**b**–**e**) the hybrids obtained by adding 0.04, 0.1, 0.2, and 0.4 g·L^−1^ graphenes in the suspension, respectively, (**f**) Cross-section of the CNT-Graphene hybrid. Note: The concentrations of CNTs and Mg^2+^ were kept certain values of 0.1 and 0.03 g·L^−1^, respectively [10].

Typical SEM images of the pristine CNTs and CNT-Graphene hybrids are exhibited in Figure 2. As shown in Figure 2a, after deposition on the SS substrate, the pristine CNTs exhibited entanglements due to the existence of MgO after heat treatment [5,10]. As shown in Figure 2b, the surfaces of the CNTs were covered by a few graphene sheets. With increasing the concentration of the graphene (Figure 2c,d), the electrode surface was mixed with more graphene sheets. When the content of graphene was increased up to 0.4 g·L^−1^ (Figure 2e), the surface was almost completely wrapped by the graphene sheets, similar results could be observed in other hybrids, e.g., CoMoO_4_ modified with chitosan [21,22]. Figure 2f illustrates the cross-section of the CNT-Graphene hybrid, showing the tightly binding properties between the CNT-Graphene hybrid of about 30 μm thick and the SS substrate.

**Figure 3:**
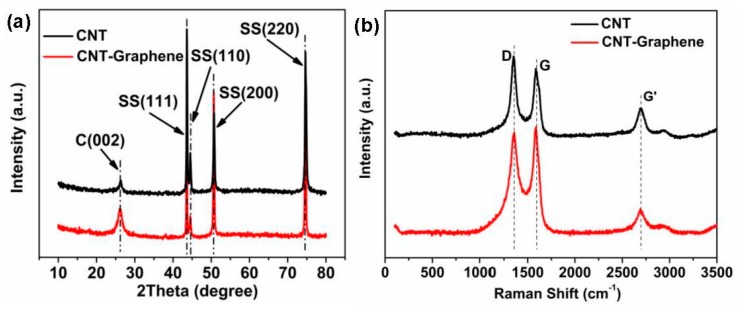
(**a**) XRD and (**b**) Raman results of the pristine CNTs and CNT-Graphene-0.1 hybrid.

XRD and Raman characterizations were applied to check the compositions of the products, as shown in Figure 3. XRD result (Figure 3a) indicates that the peak at 26.4° belongs to (002) crystallographic planes of CNT [10,11,23], and other four peaks should be attributed to the (111), (110), (200), and (220) planes of the stainless steel substrate, respectively [10,11]. As shown in Figure 3b, the Raman spectrum shows three typical characteristic peaks of the CNTs, i.e., D band (1350 cm^−1^), G band (1585 cm^−1^), and G’ (2706 cm^−1^), respectively [11,24]. The D band represents the defects of CNTs due to the existence of the disordered carbon structures in CNTs. The G band represents the graphite degree of CNTs [11]. In comparison with the pristine CNT, higher G peak from the CNT-Graphene hybrid shows better graphitization due to the introduction of the graphene. Generally, the higher the I_G_/I_D_ ratio, the better the conductivity of the sample [11,25,26]. Compared to the pristine CNTs, the higher I_G_/I_D_ ratio of the CNT-Graphene-0.1 hybrid suggests that the construction of CNT-Graphene hybrid would enhance the electrode conductivity.

### 3.2. Tensile and the Surface Resistances Tests

**Figure 4:**
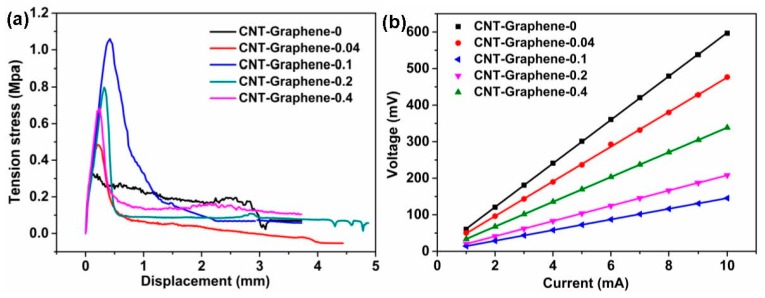
(**a**) Tensile curves and (**b**) surface resistance measurements of the pristine CNT and the CNT-Graphene hybrid electrodes.

From Figure 4a, all the CNT-Graphene hybrids exhibited higher maximum stresses than the pristine CNTs, showing clearly the adhesion improvement with the addition of graphene. Meanwhile, the maximum stress, i.e., the adhesion, of the hybrid depended highly on the graphene concentration. As shown in Figure 4b, the four-probe test showed the surface resistance of the pristine CNT electrode of about 59.6 Ω. Compared with the pristine CNT electrode, the CNT-Graphene hybrid electrodes showed lower surface resistances of 47.5, 14.6, 20.9, and 33.9 Ω, respectively, corresponding to the graphene contents of 0.04, 0.1, 0.2, and 0.4 g·L^−1^, demonstrating significant reductions of surface resistances with graphene doping. The CNT-Graphene-0.1 hybrid electrode exhibited the lowest resistance, attributed mainly to two reasons. Firstly, comparing with other grapheme doped electrodes, the CNT-Graphene-0.1 electrode presented the highest adhesion value (Figure 4a), implying the best surface contact between the hybrid and the substrate. Secondly, excessive graphene doping may lead to the agglomeration of the graphene and over-enwrapping of the CNTs with graphene sheets (Figure 2d,e), weakening the intrinsic CNT property drastically.

### 3.3. TEC Measurements

**Figure 5:**
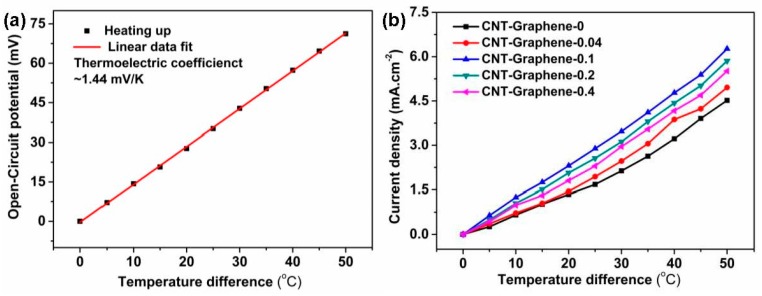
(**a**) Cell potential versus temperature difference, and (**b**) current density versus temperature difference of the pristine CNT and CNT-Graphene hybrid electrodes, each electrode surface area is 0.36 cm^2^.

The Cup-Shaped device was used to investigate the TEC performances of the pristine CNT and CNT-Graphene hybrid electrodes [5,11]. As shown in Figure 5a, by linear fitting, the thermoelectric coefficient is about 1.44 mV·K^−1^, in agreement with the previous reports for potassium ferri/ferrocyanide electrolyte [4,5,11]. For the TEC electrodes, the current densities (J_SC_) increased with the temperature differences (Figure 5b). Compared with the pristine CNT electrode, the CNT-Graphene hybrid electrodes exhibited higher J_SC_ at the same temperature difference, due to the better electrical and thermal conductivities [27,28,29] and stronger adhesion between the CNT-Graphene film and the substrate. Among all hybrid electrodes, the CNT-Graphene-0.1 exhibited the best TEC performance, with J_SC_ of 62.8 A·m^−^² and J_SC_/ΔT of 1.25 A·m^−2^·K^−1^) under a temperature difference of 50 °C.

**Figure 6:**
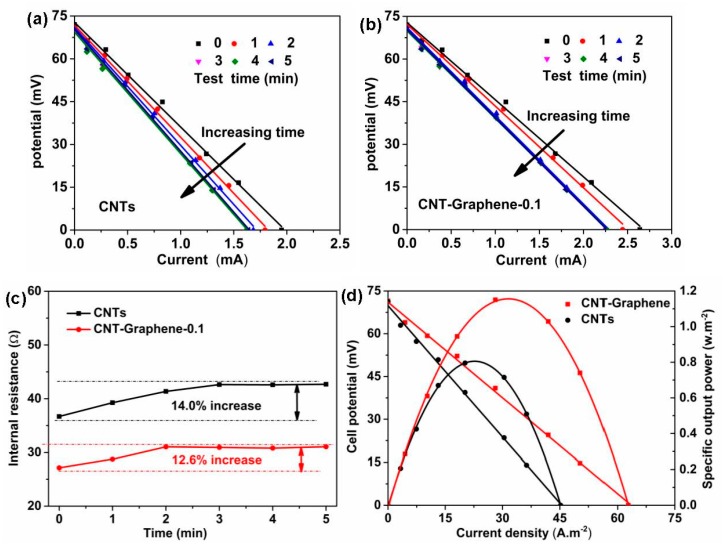
(**a**) Cell potential versus current for (**a**) CNT and (**b**) CNT-Graphene-0.1 electrodes, (**c**) fitting curve of internal resistance versus test time, and (**d**) plots of cell potential and specific output power versus current density at the steady state.

The discharge curves, the fitting relation between the internal resistance (R) with the test time, and the plots of the specific output power (P) of the TECs for the pristine CNT and CNT-Graphene-0.1 hybrid electrodes were shown in Figure 6. As shown in Figure 6a,b, the internal resistances climbed with increasing the test time for both pristine CNT and CNT-Graphene-0.1 hybrid electrodes. R could be obtained by linear fitting based on U-I curves, where U and I represented the cell potential and output current of the electrode, respectively. The output current of the electrode could be adjusted by changing the external resistance (R_ext_) according to the relation: U = V_oc_–IR, where V_oc_ is the open-circuit potential, which is mainly determined by the temperature difference [6], and R relies on the structure of the TEC [6,14,30]. For the given temperature difference, V_oc_ can be regarded as a constant, thus the relationship between U and I is approximately linear. During the continuous operation for the TEC, the built up concentration gradient, i.e., the mass transport overpotential, could be easily formed at the cold side [8,11]. The mass transport overpotential could be investigated by measuring the relation between the time and the internal resistance. As shown in Figure 6c, for the CNT-Graphene-0.1 hybrid electrode, TEC experienced a 2 min equilibrium process before obtaining a steady R, less time than the pristine CNT electrode of 3 min. In addition, the CNT-Graphene-0.1 hybrid electrode reached a steady state with R of 31.05 Ω, lower than that of the pristine CNT electrode of 42.63 Ω, attributed to the lower thermal resistance [20,27,28]. The output power is obtained based on the equation: P = UI = (V_oc_–IR) I = V_oc_ I–I^2^R, thus a quadratic relationship can be observed in the P–I curve (Figure 6d). As shown in Figure 6d, the CNT-Graphene-0.1 hybrid electrode generated J_SC_ of 62.8 A·m^−2^ and P_MAX_ of 1.15 W·m^−2^ at the temperature different of 50 °C, corresponding to a normalized current density of 1.26 A·m^−2^·K^−1^ and specific power density of 0.460 mW·m^−2^·K^−2^, respectively, 30.4% higher than those for the pristine CNT electrode, due to better conductivity and lower thermal resistance at the electrode/substrate junction [4,5,20].

The energy conversion efficiency (*η*) can be obtained using the following formula [4,8,12]:

$$\eta =\frac{0.25v_{oc}I_{sc}}{Ak(\Delta T/d)}$$

*I_sc_* and *V_oc_* are the short-circuit current and the open-circuit potential, respectively. *A* is the area of the test electrode, *k* is the thermal conductivity of the potassium ferri/ferrocyanide, ∆*T* and *d* are the temperature difference and the distance of the test electrodes, respectively. The relative energy conversion efficiency (η_r_) can be achieved by the equation: η_r_ = η/(∆T/T_h_) [4,5,11], where T_h_ is the temperature of the hot side. Based on the above equations, η_r_ of the pristine CNT electrode and CNT-Graphene-0.1 hybrid electrode reached 0.95% and 1.35%, respectively, at the temperature difference of 50 °C. Comparing with the CNT and CNT-based hybrid electrodes, the TEC performance of CNT-graphene-0.1 was moderate (See Table S1 in Supplementary Materials). Future investigations should focus on the optimization of the carbon nanomaterial with lower thermal resistance and high conductivity, e.g., single wall CNTs or doped CNTs [7,31,32]. In addition, to obtain high η_r_, it would be preferred to select the flowing type TEC to decrease further the solution diffusion resistance [4,33].

**Figure 7:**
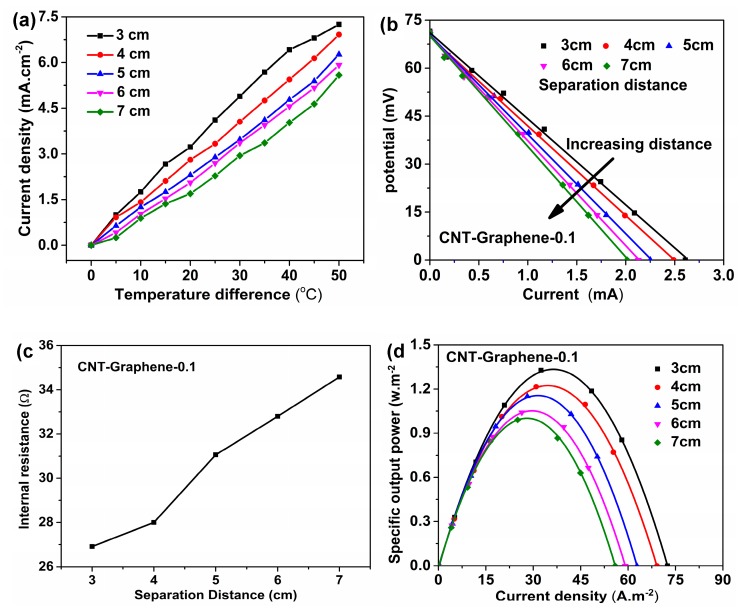
(**a**) Temperature difference versus current density, (**b**) Cell potential versus current, (**c**) Internal resistance versus time, and (**d**) plots of specific output power versus current density at different separation distances for the CNT-Graphene-0.1 hybrid electrode.

The temperature difference versus current density, the discharge curves, the fitting results of the internal resistance, and the plots of the specific output power of the TECs for the CNT-Graphene-0.1 hybrid electrode with different separation distances are shown in Figure 7. As shown in Figure 7a, with increasing the distance between the two CNT-Graphene-0.1 hybrid electrodes, the current density decreased correspondingly at the same temperature difference. In addition, the current density increased to 4.89 mA·cm^−2^ at a distance of 3 cm, 39.88% higher than that of 7 cm at the temperature different of 30 °C. Figure 7b shows different U-I curves corresponding with varying the distance, and each internal resistance can be obtained by linear fitting of the corresponding U-I curve. The values of R in Figure 7c are derived from the linear fitting of U-I curves in Figure 7b. As shown in Figure 7c, the internal resistance of the TEC increased from 26.91 Ω to 34.58 Ω, corresponding to the distance from 3 cm to 7 cm, due to extended diffusion distances of the solution [14]. As shown in Figure 7d, while reducing the separation distance of two electrodes from 7 cm to 3 cm, the specific output power could increase up to 1.33 w·m^−2^ at the distance of 3 cm, associated mainly to the drops of the internal resistance and the mass transport overpotentials [6,14]. However, the relative energy conversion efficiency decreased from 1.58 to 0.96, because the heat transport turns to be quicker thus more input thermal energy is needed to keep the same temperature difference with reducing the distance, leading to the decline of the relative energy conversion efficiency [14].

## 4. Conclusions

In this paper, CNT-Graphene hybrids were synthesized on stainless steel substrates using the electrophoretic deposition (EPD) technique to make the TEC electrodes. Such hybrid electrodes show significantly improved TEC performances in comparison with the pristine CNT electrode, due to better electrical and thermal conductivities and stronger adhesion between the CNT-Graphene hybrid electrode and the substrate. By tuning the concentrations of the graphene in the suspension, the hybrid electrodes could be optimized with the maximum current density of 62.8 A·m^−2^ and the power density of 1.15 W·m^−2^ at a temperature different of 50 °C, 30.4% higher than the pristine CNT electrode. Meanwhile, the relative conversion efficiency of 1.35% could be reached. These results suggest that the production of CNT-Graphene hybrid electrodes by EPD technique may offer a promising approach for developing CNT-based TEC electrode materials.

## Supplementary Materials

The following are available online at https://www.mdpi.com/2079-4991/9/10/1450/s1, Table S1: Comparison on TEC performances of different nanomaterials.
