# Supplementary material for: Carbon Nanotube-Graphene Hybrid Electrodes with Enhanced Thermo-Electrochemical Cell Properties
Source: Nanomaterials (Basel). 2019 Oct 12;9(10):1450. doi: 10.3390/nano9101450 (PMC6835304; doi:10.3390/nano9101450)
Supplement: Supplementary file 1 [file nanomaterials-09-01450-s001.pdf]

Supplementary Material

Carbon Nanotube-Graphene Hybrid Electrodes with Enhanced Thermo-Electrochemical Cell Properties

Yuqing Zhou, Weijin Qian *, Weijun Huang, Boyang Liu, Hao Lin, and Changkun Dong *

Institute of Micro-Nano Structures & Optoelectronics, Wenzhou University, Wenzhou 325035, China; z857744841@163.com (Y.Z.); 18857757816@163.com (W.H.); wonderain@outlook.com (B.L.); linhao.good@icloud.com (H.L.)

* Correspondence: weijinqian@wzu.edu.cn (W.Q.); dck@wzu.edu.cn (C.D.); Tel.: +86-577-8668-9067 (C.D.)

Table S1. Comparison on TEC performances of different nanomaterials.

Nanomaterial	Current density ($A.m^{-2}$)	Maximum power density ($W.m^{-2}$)	η_r	Synthesis method	Ref.
CNT	18.6	0.28	0.6	EPD	1
CNT	45.2	0.82	0.9	EPD	2
CNT buckypaper	67	1.45	1.4	Vacuum filtration	3
CNT-ACT *	0.39	$0.46*10^{-3}$	/	Dipping and drying	4
SWNT-GrO *	/	1.85	2.63	Microwave+ bath sonication	5
CNT-Pt	/	6.6	3.95	Vacuum filtration +wet chemical method	6
CNT-Ag	53.6	0.967	0.96	CVD+EPD	7
CNT-Graphene-0.1	62.8	1.15	1.35	EPD	This paper

Note: SWNT-GrO * is single-walled CNT-reduced graphene oxide; CNT-ACT * is CNT coated activated carbon textile.

References

1. Qian, W. J.; Li, M. J.; Chen, L. H.; Zhang, J. H. and Dong, C.K. Improving thermo-electrochemical cell performance by constructing Ag–MgO–CNTs nanocomposite electrodes. *RSC Adv.* **2015**, *5*, 97982-97987.
2. Qian, W. J.; Cao, M. X.; Xie, F.; Dong, C. K. Thermo-electrochemical cells based on **carbon nanotube** electrodes by electrophoretic deposition. *Nano-Micro Lett.* **2016**, *8*, 240-246.
3. Hu, R. C.; Cola, B. A.; Haram, N.; Barisci, J. N.; Lee, S.; Stoughton, S.; Wallace, G.; Too, C.; Thomas, M.; Gestos, A.; Cruz, M. E. D.; Ferraris, J. P.; Zakhidov A. A. and Baughman, R. H. Harvesting waste thermal energy using a carbon-nanotube-based thermo-electrochemical cell. *Nano Lett.* **2010**, *10*, 838-846.
4. Im, H.; Moon, H. G.; Lee, J. S.; Chung, I. Y.; Kang, T. J. and Kim, Y. H. Flexible thermocells for utilization of body heat. *Nano Res.* **2014**, *7*, 443-452.
5. Romano, M. S.; Li, N.; Antiohos, D.; Razal, J. M.; Nattestad, A.; Beirne, S.; Fang, S. L.; Chen, Y. S.; Jalili, R.; Wallace, G. G.; Baughman, R. and Chen, J. Carbon nanotube–reduced graphene oxide composites for thermal energy harvesting applications. *Adv. Mater.* **2013**, *25*, 6602-6606.

6. Im, H.; Kim, T.; Song, H.; Choi, J.; Park, J. S.; Ovalle-Robles, R.; Yang, H. D.; Kihm, K. D.; Baughman, R. H.; Lee, H. H.; Kang, T. J. and Kim, Y. H. High-efficiency electrochemical thermal energy harvester using carbon nanotube aerogel sheet electrodes .*Nat. Commun*. **2016**, *7*, 10600.
7. Zhao, F.; Qian, W. J.; Li, M. J.; Li, W.; Chen, L. H.; Zhong, F. Y.; Huang, W. J. and Dong, C. K. Directly grown carbon nanotube based hybrid electrodes with enhanced thermo-cell performances. *RSC Adv*. **2017**, *7*, 23890-23895.
